# Does a Person-Environment-Fit Promote the Academic Achievement of Hearing-Impaired Students in Malaysian Polytechnics? The Mediating Effects of Satisfaction and Adjustment

**DOI:** 10.3390/ijerph182413381

**Published:** 2021-12-19

**Authors:** Che’ Rozaniza Azizan, Samsilah Roslan, Maria Chong Abdullah, Soaib Asimiran, Zeinab Zaremohzzabieh, Seyedali Ahrari

**Affiliations:** Faculty of Educational Studies, Universiti Putra Malaysia, Serdang 43400, Malaysia; rozaniza.azizan@gmail.com (C.R.A.); mariac@upm.edu.my (M.C.A.); soaib@upm.edu.my (S.A.); z_zienab@upm.edu.my (Z.Z.); seyedaliahrari@upm.edu.my (S.A.)

**Keywords:** person-environment-fit, hearing-impaired students, adjustment, satisfaction, academic achievement

## Abstract

(1) This study investigates the influence of a person-environment-fit on academic achievement and examines mediating effects of adjustment and satisfaction on this relationship; (2) Methods: Data were collected from a sample of 195 hearing-impaired students from five polytechnics in Malaysia that offered the Special Skills Certificate program; (3) Results: Results revealed that the two constructs of the person-environment approach: personality-major fit and needs-supplies fit were positively associated with academic achievement. The adjustment was found to mediate this relationship. Taken together, these results signal that the person-environment constructs contribute to the academic achievement of hearing-impaired students and that adjustment is instrumental in elucidating this relationship; (4) Conclusions: The finding adds to the data, indicating that the person-environment-fit is a possible model of inclusion for hearing-impaired students and also provides initial data about the functioning of hearing-impaired students in Malaysian polytechnics.

## 1. Introduction

According to the Commissioner of Law Revision [1], a disabled person (PWD) is one who has extensive physical, mental, or sensory deficiencies, possibly not being able to function fully and effectively when faced with challenging situations. The World Health Organization [2] estimates that 5–10% of the world’s population is composed of people with disabilities. Based on the PWD registration data from the Department of Statistics [3] until December 2018, there were approximately 453,258 people who were registered as PWD in Malaysia. According to statistics, the hearing-impaired (HI) category stands at the 5th highest number compared to the categories of physically impaired, learning disability, mentally challenged, and visually impaired people. The term ‘hard of hearing’ is defined as those with a loss of hearing who have a lower degree of hearing compared to deafness. Over 5% of the population suffer from hearing loss disabilities [4]. The statistics provide an important implication towards the educational opportunities of HI students, as stated in the OKU Act 2008 [1]. Nevertheless, the academic outcomes for these students are far from stellar.

A study by Zadeh and Ahmadi [5] proved that the academic achievement of HI students was considerably lower as compared to the students of the same age who were not from the hard-of-hearing category. In tertiary education, Daramola et al. [6] also discovered that, despite having normal cognitive abilities, HI students’ academic achievements lag substantially below their peers of the same age. Similarly, past research has found that, despite their poorer academic achievement, HI students have several adjustment issues that impair their academic success [7,8]. The adjustment problems of HI students are more crucial than their peers who have normal hearing [9]. An initial survey carried out by the Department of Polytechnics Education [10] reported that most of these HI students had problems with learning effectively.

Ideally, students with disabilities would increase their ability to learn if their adjustment to their academic environment was strategically improved [11] (p. 17). The academic environment of a student is the primary influence on his or her learning and development [12]. These environments “reinforce and reward distinctive professional and personal self-perceptions, competencies, attitudes, interests, and values” [13] (p. 3). Holland [14] debates that behavior is the function of interactivity among the environments and individuals. Gilchrist and others [15] have stated that “picking the right learning environment is one of the most important, overwhelming, pressure-filled, confusing, and nerve-wracking ordeals a student will ever encounter” (p. 33). Students’ choices have significant implications. Inadequate fit between the student and the academic environment is likely to result in diminished satisfaction, well-being, and productivity. However, suitable matches should make polytechnic institutions less stressful and decrease the likelihood of students leaving out or transferring to different institutions. Thus, the level of student-academic environmental fit may have long-lasting effects on students’ attitudes and academic performance.

Our commitment to lowering the prevalence of inadequate student-academic environment fit has prompted us to study more about assessing the student-academic environment fit and its associated effects. The present study examined the impacts of HI student-academic environment fit using the person-environment (P-E) fit theory as a conceptual framework. The P-E fit theory is predicated on the premise that well-being and productivity are a result of people’s interactions with their environment, and that excellent fits promote well-being and lead to emotions of control, self-confidence, and contentment [16]. On the other hand, it is expected that poor fits will result in undesirable results such as unhappiness [17]. P-E fit is key to some conceptualizations of mental health: “Our fundamental concept of adjustment views adjustment as the degree of correspondence between a person’s qualities and the properties of his or her environment” [18] (p. 386).

Given the paucity of research in this area for HI students in tertiary education and recent demands for more investigation of the relationship between P-E fit and academic achievement [19], the current study examines this relationship between a sample of HI students in Malaysian polytechnics. Additionally, we seek to provide a more comprehensive view of HI students’ academic accomplishment by studying the P-E approach’s conceptions of personality-major fit, demand-abilities fit, and needs-supplies fit. The primary premise of the P-E fit theory is that it will result in positive academic outcomes [20]; nevertheless, the majority of studies have concentrated on finding the antecedent of the P-E fit instead of on its effect on academic outcomes e.g., [21]. By and large, this study has contributed to the research literature by analyzing the relationship between P-E fit and academic accomplishment, as well as whether this effect is mediated through student adaptation and satisfaction. The findings of this research will contribute to our knowledge of whether attempts to improve P-E fit can result in improved educational achievements for HI students. Hence, the purpose of the study is twofold. The first is to determine the effect of three dimensions of P-E fit (i.e., personality and major fit, demands and abilities fit, and needs and supplies fit) on the academic achievement of HI students in Malaysia. The second is to explore whether adjustment and satisfaction mediate the relationship between the dimensions of P-E Fit and the academic achievement of HI students.

### Rationale of the Study

Although much conjecture has been made about the relationship between P-E fit and academic accomplishment, little research has been conducted in the P-E fit context involving HI learners in Malaysian tertiary education, notably those enrolled in Polytechnics Special Skills Certificate [22]. There is a dearth of research on the P-E fit determinants of HI academic achievement in Malaysian tertiary education. As previously stated, the demand-abilities fit and the needs-supplies fit, which are dimensions introduced to the P-E fit, have not garnered the same level of interest as the personality-major fit [23]. No study has been conducted to our knowledge that investigates the correlations between these two characteristics of fit in HI students. Consequently, we intend to investigate these links in the current study. Furthermore, to our knowledge, no study has examined the relative impact of each of the three P-E fit subdimensions to a variety of academic-related outcomes using a complete P-E fit assessment. The current study is significant because it builds on earlier research by testing our hypothesis in a cultural context and by adding academic accomplishment as an academic-related result that was not included in their study [21,22]. As such, this study quantifies the relative amount of explained variance for each of the three fit subdimensions in academic achievement, satisfaction, and adjustment, and examines the potential differential impact of various PE dimensions on these academic-related outcomes in the Malaysian tertiary education context. Thus, we give our proposed method of the P-E fit—academic achievement relationship, followed by an explanation of the study’s unique direct and intermediary interactions.

## 2. Literature Review

### 2.1. Person-Environment (P-E) Fit Theory

As Parsons proposed in 1909, P-E fit is a crucial concept in vocational psychology. When a person’s traits and those of their environment fit, it is considered that they perform much better and are more contented. Theoretically, the higher the congruence among interests and professional environment, the better the work outcomes [24]. The concept originated with Parsons’ argument that individuals differed in their compatibility with various jobs, as well as Lewin’s field theory in the twentieth century, which asserted that behavior is a result of both individuals and their settings cited in [25]. The theory was founded on Parsons’ argument that individuals vary in their compatibility with various jobs and on Lewin’s field theory, which asserted that behavior is a result of both individuals and their circumstances [26].

To date, P-E fit has been studied extensively in several environments, including academic, groups, organizations, and individuals [16,17]. The P-E fit model is composed of three underlying constructs: personality and major fit, demands and abilities fit, and needs and supplies fit. Personality and major fit is the degree to which students’ interests and personalities fit the demands and opportunities posed by a major or program of study. The Holland hexagonal model defines the degree of fit between students’ interests and personality fit by employing the familiar six personality and environment types: artistic, realistic, social, investigative, entrepreneurial, as well as conventional (RIASEC). People, according to Holland [14], search for environments in which they can utilize their abilities and skills, convey their values, and engage in acceptable tasks.

Additionally, the needs-supplies fit takes place when an environment fulfils an individual’s needs adequately [27]; for instance, when an introverted individual is in an environment that provides adequate interpersonal space or when an individual with a strong need for accomplishment is in an environment that provides adequate opportunities for achievement. The demands-abilities fit is also influenced by an individual’s ability to meet the demands of her/his environment [28]; for instance, when individuals have the knowledge needed to complete the jobs required in a given environment. The personality-major, needs-supplies, and demands-abilities elements of fit were examined in this study as an under-researched component of P-E fit’s effect on academic achievement for HI students.

### 2.2. The Person-Environment (P-E) Fit—Academic Achievement Relationship

In their review of research on academic achievement, Nye and others [18] believe that P-E fit characteristics play a crucial role in affecting students’ academic achievement. Academic accomplishment is often expressed in terms of grade point average (GPA), which is typically calculated at the end of the semester. Thus, students who chose majors that suited their interests had a higher GPA than those who chose degrees that did not fit their interests. Nye and colleagues [18] discovered a positive correlation among congruence and performance. Individuals who enrolled in majors that were more closely linked with their interest had higher GPAs than those whose interest-major matching was less strongly associated.

Indeed, the selection of course majors that fit the personalities and abilities of students is an important quality that could help improve the academic success of the HI students [19]. In the context of course major selection, Holland [14] links personality with the extent to which individuals can adjust to their learning environments. Furthermore, past research has proven that each environment has a specific demand that is connected to the individual’s abilities and academic achievements, e.g., [20]. Academic achievement is also a representation of a student’s ability to meet environmental demands [21]. Previous research has shown that demands-ability fit and need-supply are positively associated with academic success (GPA) [20,22]. We therefore hypothesize the following:

**Hypothesis 1** **(H1a).** *Personality-major fit is positively correlated with student academic achievement*.

**Hypothesis 1** **(H1b).** *Demands-abilities fit is positively correlated with student academic achievement*.

**Hypothesis 1** **(H1c).** *Needs-supplies fit is positively associated with academic achievement*.

### 2.3. The Person-Environment (P-E) Fit in Relation to Satisfaction and Academic Achievement

Student satisfaction plays a primary role in influencing student academic achievement [24,29]. According to Cable and DeRue [30], individual satisfaction will increase significantly if the ability level required by one’s environment fulfills the actual abilities that are inherent in them. Apart from that, environmental demand that is reduced or increased will create a feeling of dissatisfaction caused either by the ability not optimally used, or demand beyond the ability of the individual [27]. In the context of education, demands-abilities fit to have a vital role in predicting the academic achievement of students in HEIs [20].

In addition, the research findings of Etzel and Nagy [20] showed that there was a significant correlation among (1) P-E fit and academic achievement; (2) P-E fit and academic satisfaction; and (3) academic satisfaction and academic achievement. Gilbreath and colleagues [28] also investigated the association between university students’ congruence and their satisfaction and psychological well-being in a study including 228 students from two mobile campuses in Indiana. The study found that, when students’ learning needs were satisfied successfully by the university, their satisfaction increased. Similar findings were obtained in a study by Li et al. [31], who stated that students’ satisfaction and academic achievement were effectively achieved when the learning environment met the students’ needs. Etzel and Nagy [20] also reported that academic achievement and satisfaction were consequences of P-E fit elements on students in Germany. Stinson [11] stated that changes in career interests were one of the key determinants for students’ inability to fulfill postsecondary education. Additionally, Stinson [11] stated that HI students were unable to select a particular academic major. As a result, these 320 HI students either dropped out of school or switched to some other academic major [32]. These results shed light on how students’ dissatisfaction can affect their academic success in tertiary education. Additionally, a path analysis of students at three Beijing universities discovered that course major satisfaction had minor mediating impact on the relationship between P-E fit and academic accomplishment [33]. According to these results, the following hypothesis was formulated:

**Hypothesis 2** **(H2a).** *Satisfaction mediates the relationship between personality-major fit and academic achievement*.

**Hypothesis 2** **(H2b).** *Satisfaction mediates the relationship between demands-abilities fit and academic achievement*.

**Hypothesis 2** **(H2c).** *Satisfaction mediates the relationship between needs-supplies fit and academic achievement*.

### 2.4. The Person-Environment (P-E) Fit in Relation to Adjustment and Academic Achievement

The concept “adjustment” refers to an individual’s process of adjusting to a new environment by overcoming stressors caused by the environment’s demands. In terms of adjustment difficulties, research indicates that HI students struggle with the social, emotional, and academic aspects of their lives [34,35]. On the premise of this view and the concepts of work adjustment theory, we propose that P-E fit has an effect on academic achievement via student adjustment. According to work adjustment theory, the more precisely a person’s abilities fulfill the needs of their function in the business, the more probable the individual will adjust effectively to work demands and achieve successful career outcomes (e.g., job satisfaction, longer tenure; [36]). According to work adjustment theory, if a person shows tenacity, flexibility, active participation, and responsiveness, this can enhance person-environment fit and adjustment to their job position [37]. Thus, in order to attain academic success, hearing-impaired pupils must be socially, emotionally, and academically attuned to the requirements of their educational environment.

Student adjustment is considered to be a predictor of academic achievement [38]. Furthermore, adjustment is indicated by a person’s personality attribute [39]. Because the perception of personality-major fit is composed of RIASEC personality qualities based on Holland’s typology theory, P-M fit has a relationship with student adjustment. We hypothesized that hearing-impaired students would experience overall adjustment in response to personality-major mismatch. This statement refers to Wessel et al. [16], who claimed that students with a lower personality-major fit, but a high level of adaptation would express higher levels of satisfaction than those with a lower level of adjustment. Moreover, previous studies have reported that demands-abilities fit and needs-supplies fit are environmental elements that may impact students’ academic achievement and satisfaction [21,40]. Thus, HI students would undergo rigorous adjustment in order to solve environmental stress induced by a mismatch between their personal (abilities and needs) and environmental (demands and supplies) factors. Once the overall adjustment is achieved, academic achievement will result in an improvement of the adjustment’s mediating effect. Therefore, the following hypotheses were formulated:

**Hypothesis 3** **(H3a).** *Adjustment mediates the relationship between personality-major fit and academic achievement*.

**Hypothesis 3** **(H3b).** *Adjustment mediates the relationship between demands-abilities fit and academic achievement*.

**Hypothesis 3** **(H3c).** *Adjustment mediates the relationship between needs-supplies fit and academic achievement*.

**Figure 1:**
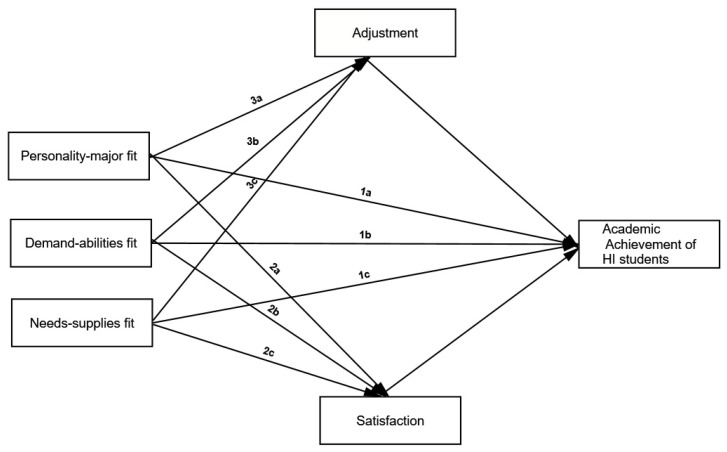
The proposed research model.

Figure 1 illustrates a proposed study model focused on the aforementioned hypotheses.

## 3. Methodology

### 3.1. Participants and Procedure

This study utilized survey design. Respondents included second- and fourth-year HI students from five Malaysian polytechnics that provided the Special Skills Certificate (SKC) program. This program involves technical and vocational training which are only specialized for HI students. When several latent variables were examined, G*Power can effectively evaluate the probability of significant associations [41]. Researchers recommended using G*Power in a PLS-SEM setting [42], and thus this work utilized G*Power version 3.1.9.7. As per Hair [42], power is set to 0.80. Participants were recruited based on their hearing range, as indicated in the PWD Registration Guideline (2009). The sample consists of all 195 participants who responded to all research variables (i.e., P-E fit, work stress, satisfaction, academic achievement, and adjustment). We noted that the results are not generalizable given the small sample size of 195. To address this, we used a quantitative approach and a participant selection process to assure that the findings were more generalizable. Quantitative approaches produce factually valid and generalizable outcomes [43]. Furthermore, in generalizing the findings of the study to similar HI students, students are randomly selected using a systematic sampling method, and we included criteria for student selection. Thus, 195 respondents were taken into account for the analysis based on the category of hearing range according to the PWD Registration Guideline (2009). HI students were split into two groups based on their hearing ability: (1) mild and moderate; and (2) severe and very severe.

Initially developed in English (see Appendix A), the questionnaire survey was translated into Malay. Given the fact that this study comprised HI students who had much poorer reading abilities compared to their hearing classmates [34], a few items were modified to account for the respondents’ reading ability. Furthermore, a visual booklet was also available to provide a clear illustration for selected items. To accommodate students with special needs, sign-language interpreters and individual assistance were also provided during the data collection. Following expert agreement on the item’s wording and context, a pilot test with 50 HI students was studied to evaluate the survey items’ clarity. No other modifications were required. All items in the final survey were presented in both English and Malay. The study was approved by the Ethics Committee of Universiti Putra Malaysia. The participants were completely voluntary and anonymous. All of the subjects gave their written informed permission. Data were collected from October 2018 to December 2018.

Table 1 shows the demographic characteristics of the participants. The majority of responses came from female students (52.4%). The majority of participants (64.8%) were Malay, 24.1% Chinese, 2.1% Indian, and 9.0% of other ethnicities. The sample also comprised students with a wide range of course majors: 52.4% majored in hotel and catering, 17.9% in graphic design, 9.7% in fashion and clothing, 13.8% in general engineering, and 6.2% in mechanical maintenance. According to hearing ability, 49% have a very severe level of hearing, 28% have a severe level of hearing, 15% have a moderate level of hearing, and 8% have mild hearing.

### 3.2. Measures

The following measures were employed to evaluate the focal constructs.

Person-environment-fit was measured using personality-major fit, demand-abilities fit, and needs-supplies fit. The Graphic-Assisted Personality-Major Congruence Instrument inventory was used in the research that originated from Self-Directed Search Form E [19], and was modified in the context of hard-of-hearing polytechnic students. This variable measured the personality of a person according to six main traits: Realistic, Social, Investigative, Enterprising, Artistic, as well as Conventional. According to a method reported by Iachan [44], personality-major fit is classified into five levels: less than 13 = weak compatibility; 14 to 19 = almost incompatible; 20 to 25 = almost compatible; and 26 to 28 = good compatibility. The Cronbach’s alpha value was 0.8.

Demand-abilities fit and needs-supplies fit were measured using questions developed in the previous study [40,45]. Demand-abilities fit was measured based on two aspects: the ability to conduct practical actions (5 items) and expectations of students’ roles in polytechnics (6 items). Needs-supplies fit was divided into two aspects: academic needs (4 items) and social needs (3 items). The continuous two-dimensional Likert Scale was used to measure this instrument, with scale 1 = agree and scale 5 = strongly agree. Cronbach’s α were 0.81 and 0.76, respectively.

Student adjustment was measured based on three main dimensions: social adjustment (8 items), emotional adjustment (3 items), and academic adjustment (10 items) [21]. The Likert Scale was also used to measure aspects of adjustment, including scale 1 = strongly unrelated and scale 5 = strongly related. The Cronbach’s alpha value obtained was 0.9.

Student satisfaction was measured using questions developed by Etzel and Nagy [20]. The variables comprised of satisfaction towards course major (5 items), satisfaction towards lecturers (3 items), satisfaction towards social support (2 items), and satisfaction towards institutions (4 items). All the questions were measured using the five-point Likert Scale; with scale 1 = strongly agree and scale 5 = strongly disagree. The Cronbach’s alpha value obtained was 0.9.

### 3.3. Data Analyses

The researchers validated the research model developed for this research utilizing PLS-SEM [46]. The authors performed the PLS algorithms with a bootstrapping fixed at 5000 subsamples using Smart-PLS version 3.2.9 data analysis software [47]. The PLS approach was selected over other regression models, since it is able to handle both the complex study model as well as the small sample size (*n* = 245), suggesting its suitability as an analytical method for this study [48]. The Standardized Root Mean Square Residual (SRMR) [49] as well as the Bentler–Bonett’s [50] Normed Fit Index were used to evaluate model fit (NFI). SRMR was used to assess the differences between actual and predicted correlation, while the NFI was used to represent the incremental measure of model fit.

## 4. Results

### 4.1. Preliminary Analysis

The means, standard deviations, Cronbach alphas, as well as bivariate correlation coefficients for the variables under study are listed in Table 2. All variables showed satisfactory skewness and kurtosis. As predicted, student adjustment, satisfaction, and academic achievement were all positively correlated with P-E fit. To assess the measuring scales’ qualities, we evaluated the reliability, convergent validity, and discriminant validity. To start, all measures showed factor loading values greater than 0.70, suggesting that every study variable showed acceptable convergent validity. All composite reliability (CR), Cronbach alpha (CA), and average variance extracted (AVE) results were higher than their respective cutoff values of 0.7 and 0.5 [50].

The Fornell–Larcker as well as Heterotrait–Monotrait (HTMT) criteria were used to assess discriminant validity, as suggested by Fornell and Larcker [51] and Henseler et al. [52]. As per the Fornell–Larcker criterion, the square root of every construct’s AVE was higher than the correlations of all the other constructs. The results of the Heterotrait–Monotrait (HTMT) analysis were well within the prescribed limit of 0.85 (range 0.359 to 0.846) [51].

### 4.2. Hypothesis Testing

#### 4.2.1. Structural Model

**Figure 2:**
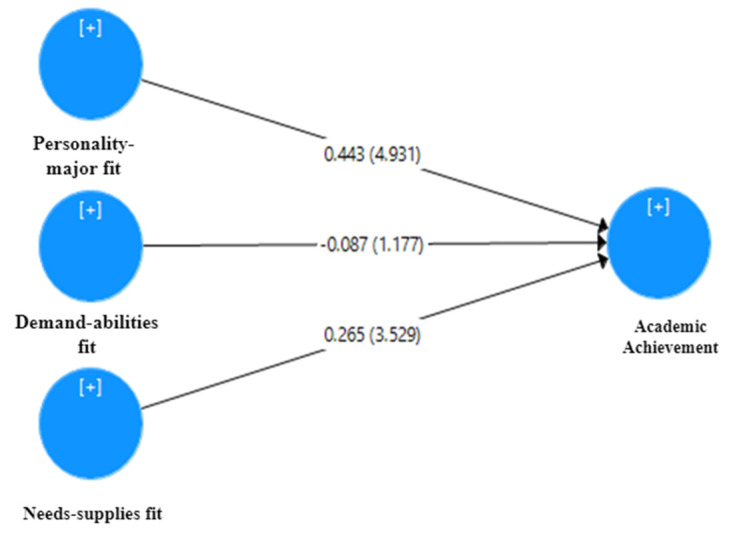
The structural model for academic achievement.

The significance of path coefficients, effect size (f^2^), coefficient of determination (R^2^), and predictive relevance of the structural model were evaluated (Q^2^). The findings confirm that the data fit the model well, with SRMR values less than 0.026 and NFI values greater than 0.98 for all models [52]. Academic achievement had an R^2^ of 0.271, indicating a weak-to-moderate relationship [52]. Collinearity was measured by calculating the VIF values, which were all less than six for all constructs used in the analysis, indicating that collinearity did not indicate a risk. The findings suggest that the portion of academic achievement predicted by needs-supplies fit is significant (=0.269, 3.529, *p*-value = 0.000). Furthermore, the route coefficients show a connection between the personality-major fit (=0.443, t = 4.931, *p*-value = 0.000) and academic accomplishment. Furthermore, no significant relationship between demand-abilities fit and academic achievement was discovered (=−0.087, t = 1.177, 0.24). As illustrated in Figure 2, these results do not rule out H_1a_ and H_1c_.

Henseler [52] f^2^ classification used in this study, with 0.02 being small, 0.15 indicating medium, and 0.35 indicating large. The f^2^ scores for demand-abilities fit and needs-supplies fit were 0.156 and 0.178, respectively, while the personality-major fit value was 1.194. This demonstrated measures in explaining academic attainment. Moreover, this research used Q^2^ to estimate the predictive significance of job burnout as an endogenous variable. According to Henseler et al. [52], the Q^2^ classifications are 0.02 for small, 0.15 for medium, and 0.35 for large. The Q^2^ result was also 0.249, suggesting that the academic achievement had a strong predictive ability.

#### 4.2.2. Mediation Tests of Student Adjustment and Satisfaction

A bootstrapping technique was utilized to assess the proposed model of mediation [53]. As a consequence, a sample of 5000 students was selected randomly with replacement from the original data to assess the indirect relationship among P-E fit and academic achievement, as mediated by student adjustment, as well as satisfaction. To assess parallel mediation, the bootstrapping method was applied, which involves conducting a resampling procedure with a substitution that has no relation to the normal distribution of the data [53]. The results (see Table 3) revealed that student adjustment played a partially significant mediating role in the relationship among needs-supplies fit (=0.32, t = 3.337, *p* = 0.001), personality-major fit (=0.441, t = 5.48, *p* = 0.000), and academic achievement. Furthermore, student adjustment (=0.212, t = 3.055, *p* = 0.02) fully mediated the relationship among demand-ability fit and academic accomplishment. Moreover, the results revealed that student satisfaction did not act as a mediator between the P-E fit and academic achievement constructs (see Table 2). H_2a_, H_2b_, and H_2c_ were thus supported. Indirect models explained on average 34%, 36.1%, and 46.8% of the variation in academic achievement, student adjustment, and satisfaction, respectively.

## 5. Discussion and Implications

In response to recent calls to evaluate whether three aspects of P-E fit, namely personality major fit, ability-demands fit, and need-supplies fit, correlate with the enhanced academic achievement of HI students in polytechnics, we evaluated an integrative model in which P-E fit environment predicts academic satisfaction via two primary mechanisms: improved student adjustment and satisfaction. Consistent with Holland’s theory [14], we found that personality major fit is strongly linked to the academic achievement indicated by HI students. Smart and Umbach [54] consistently found that the students focus on academic environments that fit their personality types and that those environments promote and reward a specific set of attitudes and talents. They concluded that students whose personality types match their academic environment are more likely to exhibit the attitudes and abilities valued in that environment. Academic achievements (grade levels), the majority of which are issued by faculty members who are actively engaged in the academic environment, are indeed clear rewards for good behavior. Rocconi et al. [55] also found that P-E fit is positively related to self-reported grades. Previous research found that students make major choices based on personal and environmental factors [29]. It was also found that students begin to perform poorly when they lose or do not have interest in their major [30].

The study’s findings confirmed that HI students thriving in environments that are suited for their personality types have significant implications for their learning and success in polytechnics. Therefore, a narrative approach can be developed that encourages HI students to adapt to their study environment’s personality type through the creation of alternative and more active plots for their academic environment scenarios. Likewise, the construction interview has been recommended as a technique for strengthening one’s sense of meaning in their work by creating academic environment scenarios that connect one’s self-identity to their work role. Besides the narrative approach, educational authorities, especially the Department of Polytechnic Education, should consider instituting a specialized academic major selection system to address issues of personality and academic major incompatibility. This allows a more thorough and systematic strategy to academic major selection, resulting in a more favorable student outcome. Moreover, counsellor assistance is believed to enable appropriate career planning, followed by suitable treatment to fulfill the educational needs of hearing-impaired students.

While the results did not support the association between ability-demands fit and academic achievement, they did support the relationship between need-supplies fit and academic achievement. This finding is consistent with a previous study that found that excess supplies can be used to fulfill students’ other values, thus improving students’ academic achievement [28]. Positive correlations between social, academic, and physical environment supplies confirm this. Therefore, when both needs and supply were high, HI students’ academic achievement at their polytechnic schools was significantly higher than when both were low. This suggests that polytechnics should consider what HI students want and how much they need from academic settings. The fulfilment of higher-valued needs was associated with positive outcomes in our sample. Besides extracurricular training sessions, such as those on learning strategies, HI students will benefit from improving the abilities necessary for their program of study and thus improving objective fit. As our results suggest, individual abilities that exceed situational requirements can potentially be negative. Additional programs (i.e., increased demand) targeted to specific students can help bridge that gap. For instance, such students may be accepted to a special program, earn additional credentials, or be given additional research responsibilities. To conclude, not only can students’ talents be tailored to meet the demands, but also the learning environment.

Additionally, the findings revealed that student adjustment seems to mediate the impact of P-E fit on academic achievement. Many earlier research studies have shown that student adjustment is a significant predictor of academic outcomes [56]. Moreover, without an awareness of the possible role of P-E fit, the critical mediator role of student adjustment was confirmed. It seems especially relevant to us to acknowledge the role of student adjustment, as this leads the way for the development of support programs in tertiary education institutions that are explicitly focused on HI students in polytechnics. Since it is generally easier to change the environment or the job than it is for HI students, the majority of this group of students had to cope with impairments that were not susceptible to major change. Together with highlighting opportunities available within the academic setting, counsellors should carefully study and highlight the setting’s characteristics in order to make necessary adjustments.

Nevertheless, we discovered no evidence of an association among physical fitness and academic achievement through the mediating impact of satisfaction. Perhaps this is not totally unexpected. While the relationship between person-environment congruence and satisfaction has long been a core component of the P-E fit literature [14], a few have suggested that this relationship is not as robust as previously suspected [57]. For instance, a meta-analysis of person-environment congruence [58] that used the Holland model for assessing interests and environments found evidence of a large impact of size variability among trials. The authors of the study found a mean correlation of 10 between physical fitness and satisfaction in the college setting. Furthermore, Bai and Liao [58] found that environments with high investigative codes, comparable to the one under inquiry, demonstrated below-average relationships between satisfaction and P-E fit. Furthermore, Harackiewicz et al. [59] showed that not all outcomes are correlated with a specific type of fit. If fitting into an environment requires an open, academic mindset, we might anticipate that fit is related to academic performance, as measured by grades and awards. However, if fitting in had little or no bearing on the process’s enjoyment, one would not anticipate fit to be associated with satisfaction in that context. Furthermore, the study’s findings support the notion that student satisfaction is a poor predictor of academic progress. When fit relationships were investigated in this research, it was found out that the majority of personality–environment combinations that strongly predicted pleasure did not predict HI students’ achievement.

In summary, our results significantly contribute to the ongoing information on P-E fit and the academic achievement of students with hearing disabilities. Much of the academic achievement literature has focused on non-academic environment-related determinants of academic achievement, prompting calls for a more thorough evaluation of how environmental factors interact with student-level individual differences to influence academic success [60]. We were able to gain an in-depth understanding into how students’ abilities, demands, and personality differences in the tertiary educational setting, and academic outcomes, shape perceptions of academic achievement by testing our model, which attempts to measure three constructs of P-E fit and different components of academic outcomes (i.e., student adjustment and satisfaction). Taken altogether, our findings establish the P-E fit components as predictors of academic achievement and imply that processes associated with enhanced adjustment and satisfaction may help to explain how P-E fit students in polytechnics report higher academic achievement.

## 6. Limitations and Future Research Directions

This study applied a cross-sectional design. According to Schmitt et al. [61], P-E fit is a continuous process that develops with the subject’s maturity and experience. As a result, an in-depth analysis of students’ adjustment movements across semesters is feasible. Furthermore, further research can be done using a longitudinal approach to identify the differences in students’ personalities, abilities, and needs over the course of their polytechnic studies. According to the findings of the study, the indirect model accounts for up to 34% of HI students’ academic achievements. Future researchers are recommended to examine additional factors that may contribute to HI students’ academic achievement at polytechnics, such as family factors, sign language use, psychosocial factors, communication factors, and the role of hearing aids, in order to predict their academic achievement. Moreover, this survey only included HI students enrolled in Special Skills Certification courses at Malaysian polytechnics. Further research is recommended to expand the focus of the research to include all tertiary institutions such as PWD-related skills training institutes, private colleges, teacher training centers, and universities. The research results can also be extended to include the entire population of HI-enrolled students in all Malaysian tertiary institutions.

## 7. Conclusions

In conclusion, Person-Environment (P-E) Fit Theory is an effective theory that can be employed to investigate the relationship between person-environment-fit and academic achievement of hearing- impaired students in Malaysian polytechnics. The results contribute to educational psychology literature by providing additional empirical support for the capacity of the two constructs of the person-environment-fit (i.e., personality-major fit and needs-supplies fit) in predicting academic achievement. The results also indicate that student adjustment mediates the mentioned person-environment-fit constructs and academic achievement. It is hope that educators will make use of these constructs for facilitating environmental change in an attempt to enhance academic achievement of hearing-impaired students.

## Figures and Tables

**Table 1:** Demographic characteristics of the respondents.

Respondents		Frequency	Percent (%)
Gender	Male	93	47.6
	Female	102	52.4
Age	19–20 years old	83	42.4
	21–22 years old	105	53.8
	>23 years old	7	3.8
Nationality	Malay	126	64.8
	Chinese	47	24.1
	Indian	4	2.1
	Others	18	9
Academic major	Hotel and catering	102	52.4
	Graphic design	35	17.9
	Fashion and clothing	19	9.7
	General engineering	27	13.8
	Mechanical maintenance	12	6.2
Hearing-ability level	Minimum/Mild (20–30 dB)	16	8
	Moderate (30–60 dB)	29	15
	Severe (60–90 dB)	55	28
	Very Severe (>90 dB)	96	49

**Table 2:** Descriptive statistics and bivariate correlations.

Construct	Mean	SD	1	2	3	4	5
1. Academic achievement	3.384	0.382					
2. Satisfaction	5.613	0.837	1				
3. Student adjustment	7.917	0.727	0.519 **	1			
4. Personality-major fit	3.386	0.421	0.644 **	0.488 **	1		
5. Demand-abilities fit	6.075	0.876	0.553 **	0.573 **	0.562 **	1	
6. Needs-supplies fit	8.637	0.704	0.258 **	0.186 *	0.256 **	0.113	1

Note: *N* = 245; **, * significant at alpha =*p* < 0.01.

**Table 3:** Mediation analysis.

Paths	Total Effect		Direct Effect		Indirect Effect				Bias Corrected Bootstrap (95% CI)	
	Coefficient	*p*	Coefficient	*p*	Paths	Coefficient	t-Value	*p* Values		
Demand-abilities fit → Academic achievement	−0.087	0.252	−0.254	0.005	Demand-abilities fit → Satisfaction → Academic achievement	0.024	0.702	0.483	−0.047	0.084
Needs-supplies fit → Academic achievement	0.265	0	0.23	0	Needs-supplies fit → Satisfaction → Academic achievement	0.009	0.57	0.569	−0.015	0.048
Personality-major fit = P-M fit → Academic achievement	0.443	0	0.333	0	Personality-major fit = P-M fit → Satisfaction → Academic achievement	0.038	0.691	0.49	−0.057	0.159
					Demand-abilities fit → Adjustment → Academic achievement	0.144	3.055	0.002	0.011	0.18
					Needs-supplies fit → Adjustment → Academic achievement	0.32	3.337	0.001	0.107	0.482
					Personality-major fit = P-M fit → Adjustment → Academic achievement	0.441	5.48	0.000	0.252	0.595

## Appendix A

**Table A1:** The questionnaire’s items.

Construct	Measurement Item
**Student satisfaction**	I feel satisfied to choose this field of study.
	I have no desire to change my field.
	I am passionate about this field.
	I am interested in this field.
	I feel satisfied with the learning activities in the classroom.
	I feel satisfied with the manner in which the instructors teach.
	I feel satisfied because the instructor is proficient in sign language.
	I feel satisfied because the instructor does not treat me differently.
	I feel satisfied with the co-curricular activities at the polytechnic.
	I feel satisfied with the peer coaching activities at the polytechnic.
	I feel satisfied with classroom support services such as sign language interpretation.
	I feel satisfied with the help by friends in the classroom.
	I feel satisfied with the OKU-friendly facilities (e.g., washroom condition, rooms, cafeteria).
	I feel satisfied with the learning facilities at the Polytechnic.
**Adjustment**	I make good friends with my classmates.
	I have a good relationship with my instructor.
	I participate actively in group discussions.
	I participate in all of the activities planned by the Special Skills Certificate instructors.
	I keep track of the activities at the polytechnic.
	I keep track of the Polytechnic’s activities with friends, without hearing impairments.
	I participate regularly in extra-curricular activities.
	I do not have difficulty in interactioning with my classmates.
	I am not alone at the Polytechnic.
	I make friends who do not have hearing impairments.
	My instructor is someone I can count on to assist me.
	I seek the help of the Student Counseling Unit when confronted with a problem.
	I have a good sense of self-adjustment at the Polytechnic.
	I solve problems on my own.
	I have never skipped a class or a practical session.
	If I do not understand what is being taught, I meet with the instructor for assistance.
	Despite the difficulties encountered, I shall attempt to finish the instructor’s assignments.
	Despite my hearing impairment, I attend practical training courses.
	The instructor helps me improve the quality of my practice.
	I took tutorial classes to improve my overall understanding.
	If needed, I will ask the instructor to schedule additional classes for me.
**Academic chievement**	I often repeat a year or carry modules over to the next academic year/semester.
	Since starting polytechnic studies, I have never ever failed an examination.
	I have performed well in my past semester examinations.
	I am good in most of my modules.
	I am able to achieve the academic goals that I have set.
**Demand-abilities fit**	The assignments given by the instructors were suitable for my sign language abilities.
	My skills and knwledge are a good match for the assignments given by the instructors.
	My ability to understand assignments given by instructors is adequate.
	The time given by the instructors to perform the assignment is sufficient.
	I believe that studying at the polytechnic is an exciting opportunity that is an excellent match for my hearing impairment.
	My abilities and training are a good fit with the requirements of studying at the polytechnic.
	The match is very good between the demand for graduates and programs at the polytechnic.
	The demand of my time to complete my studies and the length of my studies at the polytechnic are perfectly suited.
	I have the skills to undergo practical training successfully.
	The practical training in the workshop suited my hearing disability.
	My personal abilities provide a good match with the demands of the practical training.
**Needs-supplies-fit**	There is a decent fit between the instructor’s notes and the notes I need for my study.
	The academic achivement that I look for are fulfilled very well by my efforts.
	The availability of an instructional instrument (computers, LCDs, etc.) in the classroom is exactly what I need for my learning.
	The teaching techniques used by instructors can help me to improve my learning.
	Ihe sign language used by the instructors is similar to the sign language I am familiar with.
	My instructor provides me with the praise that I seek in the classroom.
	The responsibilities that were given to me (e.g., being a class leader, a representative of the Student Representative Council, etc.) make me feel valued and cherished.
**Personality-major fit**	This section contains graphic and visual aids (pictures) for the items of the personality-major fit that can be used as a student reference.
